# Comparison of Photopic Negative Response of Full-Field and Focal Electroretinograms in Detecting Glaucomatous Eyes

**DOI:** 10.1155/2011/564131

**Published:** 2010-09-30

**Authors:** Shigeki Machida, Kunifusa Tamada, Taku Oikawa, Yasutaka Gotoh, Tomoharu Nishimura, Muneyoshi Kaneko, Daijiro Kurosaka

**Affiliations:** Department of Ophthalmology, School of Medicine, Iwate Medical University, 19-1 Uchimaru Morioka, Iwate 020-8505, Japan

## Abstract

*Purpose*. To compare the photopic negative response (PhNR) of the full-field electroretinogram (ERG) to the PhNR of the focal ERGs in detecting glaucoma. *Methods*. One hundred and three eyes with glaucoma and 42 normal eyes were studied. Full-field ERGs were elicited by red stimuli on a blue background. The focal ERGs were elicited by a 15° white stimulus spot centered on the macula, the superotemporal or the inferotemporal areas of the macula. *Results*. In early glaucoma, the areas under the receiver operating characteristic curves (AUCs) were significantly larger for the focal PhNR (0.863–0.924) than those for the full-field PhNR (0.666–0.748) (*P* < .05). The sensitivity was significantly higher for the focal PhNR than for the full-field PhNR in early (*P* < .01) and intermediate glaucoma (*P* < .05). In advanced glaucoma, there was no difference in the AUCs and sensitivities between the focal and full-field PhNRs. *Conclusions*. The focal ERG has the diagnostic ability with higher sensitivity in detecting early and intermediate glaucoma than the full-field ERG.

## 1. Introduction

It has been generally believed that the activity of retinal ganglion cells (RGCs) contributes little to shaping the corneal electroretinogram (ERG) elicited by ganzfeld stimuli (full-field ERG). However, a response has been newly identified to originate from RGCs that receive signals from cones [1]. This response was termed the photopic negative response (PhNR) [2], and it consists of a negative-going wave that follows the photopic cone b-wave. 

 The PhNR is strongly attenuated in primate's eyes with experimentally induced glaucoma and also in eyes intravitreally injected with tetrodotoxin [2], a blocker of the neural activity of retinal ganglion cells, their axons, and amacrine cells [3, 4]. In addition to this experimental evidence, it has been demonstrated that the PhNR was reduced in patients with optic nerve and retinal diseases that affect mainly the RGCs and retinal nerve fiber layer [5–16]. We have shown that the amplitudes of the PhNR of the full-field cone ERG (full-field PhNR) were correlated with visual sensitivity, disc topography, and retinal nerve fiber layer thickness in eyes with open angle glaucoma (OAG) [16]. These results indicate that the full-field PhNR can be used as an objective functional measure of the RGCs in glaucomatous eyes.

When the full-field PhNR amplitude was used as a diagnostic tool, the sensitivity and specificity to discriminate glaucomatous from normal eyes were 77% and 90%, respectively [16]. However, at the early stage of glaucoma, the sensitivity was reduced to 57%, indicating that the full-field PhNR is not suitable for diagnosing early glaucoma. This is not surprising because the early glaucomatous changes begin with localized neuronal loss in the retina and optic nerve head that could not be detected by the full-field ERG. 

The focal ERG system originally developed by Miyake et al. [17] is now commercially available in Japan. Recently, we have recorded focal ERGs from patients with glaucoma [18–20] and optic nerve diseases [21]. We found that the PhNR of the focal ERG (focal PhNR) was also selectively attenuated in patients with OAG. In addition, we investigated correlation between the focal PhNR and corresponding retinal sensitivity obtained by standard automated perimetry (SAP). A curvilinear relationship was found between the focal PhNR amplitude and retinal sensitivity (decibel), in which a reduction of the focal PhNR amplitude was associated with a small decrease of retinal sensitivity at the early stage of glaucoma [18]. This suggests that the focal PhNR may be used for detecting functional loss at the early stage of glaucoma. This focal ERG system allows us to record focal retinal responses from the paramacular regions of the retina that are preferentially affected at the early stage of glaucoma. In our recent study, we recorded focal ERGs from three retinal loci including the macular region, the supero-temporal and infero-temporal areas of the macula. The sensitivity and specificity of the focal PhNR to discriminate early glaucoma were >90%. These findings were made with the combined criterion in which eyes were classified as being glaucomatous when the focal PhNR amplitudes were less than the optimal cut-off values in either retinal locus [19].

From these results, it appeared that the focal PhNR is better than the full-field PhNR to discriminate glaucomatous from normal eyes. However, a direct comparative study comparing the diagnostic values of full-field and focal PhNRs obtained from the same eyes has not been reported although studies using different patient populations for the full-field and focal PhNRs have been done [16, 18, 19].

Thus, the purpose of this study was to compare the ability of the full-field and focal PhNRs to detect glaucomatous eyes at different stages. Importantly, the full-field and focal PhNRs were recorded from the same eyes.

## 2. Methods

### 2.1. Patients

One hundred and three eyes of 103 patients with OAG were studied. Their ages ranged from 37 to 83 years with a mean ± standard deviation of 68.2 ± 9.1 years. The diagnosis of OAG was based on the presence of a glaucomatous optic disc associated with visual field defects measured by SAP. The presence of glaucomatous optic disc was determined by the guideline of Japanese Society of Glaucoma developed in 2005 (http://www.nichigan.or.jp/member/guideline/glaucoma2.jsp). According to the diagnostic criterion for minimal abnormality of the visual field [22], the visual field defect was determined to be glaucomatous when it met one of three criteria. (1) The pattern deviation plot showed a cluster of three or more nonedge points that had lower sensitivities than those in 5% of the normal population (*P* < .05), and one of the points had a sensitivity that was lower than 1% of the population (*P* < .01), (2) the value of the corrected pattern standard deviation was lower than that of 5% of the normal visual field (*P* < .05), or (3) the Glaucoma Hemifield Test showed that the field was outside the normal limits.

Forty-two eyes of 42 age-matched normal volunteers, ranging in age from 53 to 78 years with a mean of 67.6 ± 7.3 years, were studied. We selected normal eyes from patients with macular hole in the fellow eye which was treated by vitrectomy. They underwent comprehensive ophthalmological examinations including measuring visual acuity by a Snellen chart and observing the ocular fundus by an indirect ophthalmoscope as well as a biomicroscopic slit lamp. In addition, we performed optical coherence tomography and SAP to rule out macular and optic nerve diseases.

This research was conducted in accordance with the Institutional Guidelines of Iwate Medical University, and the procedures conformed to the tenets of the Declaration of Helsinki. An informed consent was obtained from all subjects after a full explanation of the nature of the experiments.

### 2.2. ERG Recordings

The pupils were maximally dilated to approximately 8 mm in diameter following topical application of a mixture of 0.5% tropicamide and 0.5% phenylephrine HCL. The recordings of the full-field and focal ERGs were made on the same eye on the same day. The stimulus conditions for the recordings of the full-field cone ERGs and focal ERGs have been reported in detail [16, 18].

The full-field cone ERGs were elicited by red stimuli of 1 600 cd/m^2^ (*λ*_max_ = 644 nm, half-amplitude bandwidth = 35 nm) on a blue background of 40 cd/m^2^ (*λ*_max_ = 470 nm, half-amplitude bandwidth = 18 nm). The duration of the stimulus was 3 msec. The stimulus and background lights were produced by light emitting diodes (LEDs) embedded in the contact lens. 

**Figure 1:**
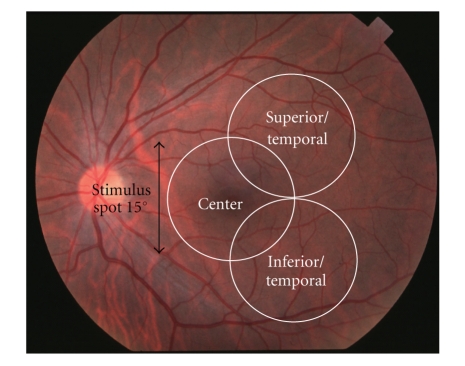
Ocular fundus photograph showing retinal areas which were stimulated by focal spots with a diameter of 15 degrees.

Focal ERGs were recorded from the macular area and from the supero-temporal and infero-temporal areas of the macula. Responses from these areas are designated as the center, superior/temporal, and inferior/temporal responses, respectively (Figure 1). The stimulus system was integrated into the infrared fundus camera (Mayo Co., Nagoya, Japan), which had been developed by Miyake et al. [17]. The stimulus spot was 15 degrees in diameter and was placed on the retinal area of interest, and the position was confirmed by viewing the ocular fundus on a monitor. The intensity of the white stimulus and background lights was 165 cd/m^2^ and 6.9 cd/m^2^, respectively. The stimulus duration was 10 ms. The focal ERGs were recorded with a Burian-Allen bipolar contact lens electrode (Hansen Ophthalmic Laboratories, Iowa City, IA). 

**Figure 2:**
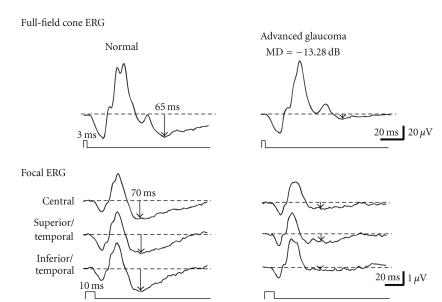
Representative full-field cone and focal electroretinograms recorded from a normal subject and a glaucoma patient with advanced visual field defects.

The responses were digitally band-pass filtered from 0.5 to 1000 Hz for the full-field ERG and from 5 to 500 Hz for the focal ERG. It is often difficult to determine the negative trough of the PhNR especially in cases with reduced PhNR amplitudes. Therefore, we measured the PhNR amplitude at the fixed time points. We determined the time of the maximum amplitude of the PhNR in normal subjects according to the method of Rangaswamy et al. [9]. We found that the full-field and focal PhNRs were the largest at 65 ms and 70 ms after the flash, respectively. Therefore, we measured PhNR amplitudes at 65 ms for the full-field PhNR and 70 ms for the focal PhNR throughout the study (Figure 2).

### 2.3. Visual Field Analyses

The Humphrey Visual Field Analyzer (Model 750, Humphrey Instruments, San Leandro, CA, USA) was used for SAP. The SITA Standard strategy was applied to program 24-2. From the mean deviation (MD) of the 24-2 program, we classified patients with glaucomatous visual fields into three groups: early (MD >−6 dB; *n* = 41, mean age and SD: 68.6 ± 9.8 years), intermediate (−6 dB ≥ MD ≥−12 dB; *n* = 28, 69.5 ± 8.1 years), and advanced (MD <−12 dB; *n* = 34, 69.4 ± 7.4 years) defects of the visual field. There was no significant difference in the mean age among the three groups. The intraocular pressures (IOPs) of all patients were controlled under 21 mmHg by eye drops, and there was no significant difference in the IOPs among the groups. The averaged MDs were −3.31 ± 1.58, −8.88 ± 1.67, and −17.37 ± 4.46 dB for the early, intermediate, and advanced groups, respectively.

When the fixation loss rate is higher than 20%, the field examination was determined to be unreliable and excluded from the analysis. In addition, when the false-positive or false-negative error rates exceeded 33%, the visual field was not used for the analysis. The interval between the visual field testing and ERG recording was less than 1 month.

### 2.4. Statistic Analyses

We used receiver operating characteristic (ROC) curves to determine the optimal cut-off values that yielded the highest likelihood ratio. The area under the curve (AUC) was used to compare the ROC curves. The comparison between AUCs was made according to the method reported by DeLong et al. [23]. The sensitivity and specificity of the focal PhNR were compared to that of the PhNR of the full-field ERGs using Fisher's exact test. Unpaired *t* tests were used to compare data between groups with different degrees of the visual field defect. One-way ANOVA was used to determine the statistical significance of the ERG changes in eyes with the stage of glaucoma. These analyses were performed using commercial software MedCalc 11.3.3 (MedCalc Software, Mariakerke, Belgium) and Prism 5.1 (GraphPad Software Inc., San Diego, CA).

## 3. Results

### 3.1. Representative ERG Waveforms from Normal and Glaucomatous Eyes

The full-field and focal ERGs recorded from a normal control and a patient that had advanced glaucoma with a mean deviation −13.28 dB are shown in Figure 2. Both the full-field and focal PhNRs were reduced in the patient compared to the normal control although there was no change in the amplitudes of the a- and b-waves in the full-field and focal ERGs (Figure 2).

### 3.2. Averaged PhNR Amplitudes and PhNR/b-Wave Amplitude Ratios for Different Degrees of Visual Field Defects

**Figure 3:**
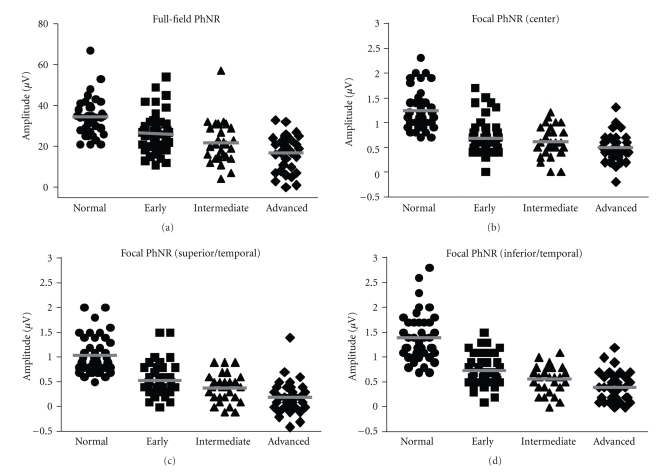
The PhNR amplitudes of the full-field (a) and focal ERGs (b) center, (c) superior/temporal, and (d) inferior/temporal) are plotted for the normal controls (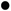) and glaucomatous eyes at early (■), intermediate (▴), and advanced stages (*◆*). Bars represent means of the PhNR amplitudes.

**Figure 4:**
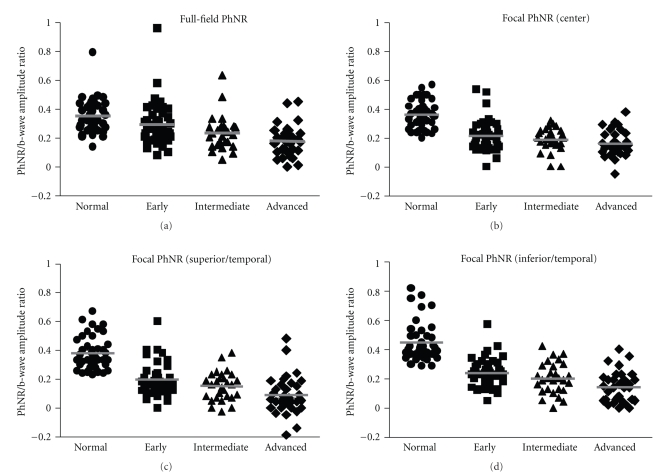
The PhNR/b-wave amplitude ratios of the full-field (a) and focal ERGs (b) center, (c) superior/temporal, and (d) inferior/temporal) are plotted for the normal controls (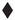) and glaucomatous eyes at early (■), intermediate (▴), and advanced stages (*◆*). Bars represent means of the PhNR/b-wave amplitude ratios.

We have plotted the PhNR amplitudes and PhNR/b-wave amplitude ratios against stages of glaucoma in Figures 3 and 4, respectively. In both the full-field and focal ERGs, the PhNR amplitudes and the PhNR/b-wave amplitude ratios were significantly and progressively reduced with an advance in the stage of glaucoma (*P* < .0001). Even at the early stage of glaucoma, the PhNR amplitude and PhNR/b-wave amplitude ratio were significantly reduced compared to that in the normal controls for the full-field (PhNR amplitude: *P* < .004) and focal ERGs (all retinal areas: *P* < .0001). However, for the PhNR/b-wave amplitude ratio of the full-field ERGs, the data of the normal control considerably overlapped those from the early glaucoma group resulting in no significant differences (Figure 4(a)).

The PhNR amplitude and PhNR/b-wave amplitude ratio of the full-field ERGs gradually decreased as the stage of glaucoma advanced. On the other hand, the greatest loss of the PhNR amplitude and PhNR/b-wave amplitude ratio of the focal ERG was seen at the early stage of glaucoma. For example, the mean of the focal PhNR amplitude recorded from the center was reduced from 1.24 *μ*V to 0.69 *μ*V at the early stage of glaucoma. Then, it slightly decreased to 0.50 *μ*V at the advanced stage of glaucoma despite considerable loss of the visual sensitivity of SAP (Figure 3(b)).

The full-field PhNR amplitude fell outside the normal range in 29, 48, and 56% of patients of the early, intermediate, and advanced groups. The focal PhNR amplitudes of the central retinal area fell outside the normal range in 62, 61, and 76% of patients of the early, intermediate and advanced groups. The corresponding percentages for the superior/temporal and inferior/temporal focal PhNR amplitudes were 49 and 46% for the early, 59 and 57% for the intermediate, and 85 and 79% for the advanced groups, respectively. Thus, the focal PhNR amplitude showed abnormal values in more patients at any stages than the full-field PhNR amplitude. Similar results were obtained for the PhNR/b-wave amplitude ratio.

### 3.3. ROC Curves of Full-Field and Focal ERGs

**Figure 5:**
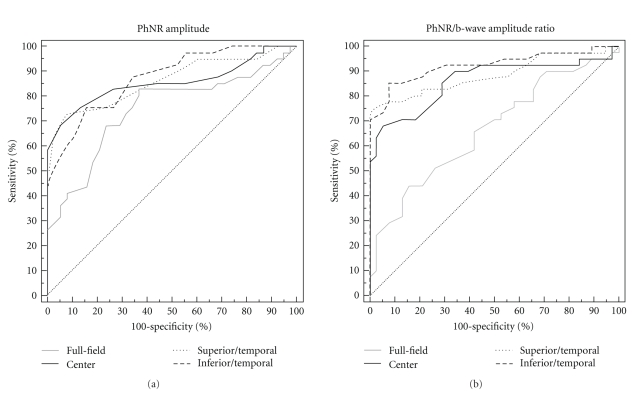
Receiver operating characteristic (ROC) curves for the PhNR amplitude (a) and PhNR/b-wave amplitude ratio (b) of the full-field and focal electroretinograms. Patients with early glaucoma (*n* = 41, mean deviation >−6 dB). PhNR: photopic negative response.

**Figure 6:**
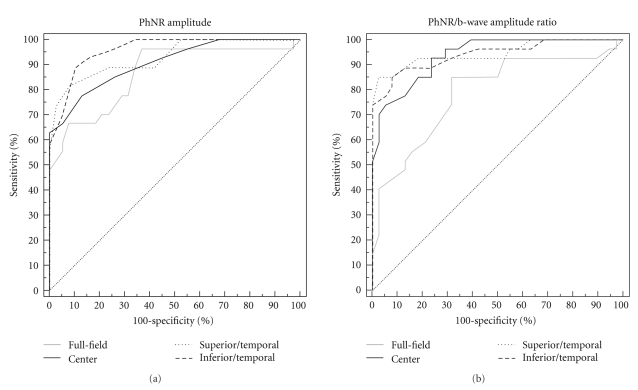
Receiver operating characteristic (ROC) curves for the PhNR amplitude (a) and PhNR/b-wave amplitude ratio (b) of the full-field and focal electroretinograms. Patients with intermediate glaucoma (*n* = 28, −6 dB ≥ mean deviation ≥−12 dB). PhNR: photopic negative response.

**Figure 7:**
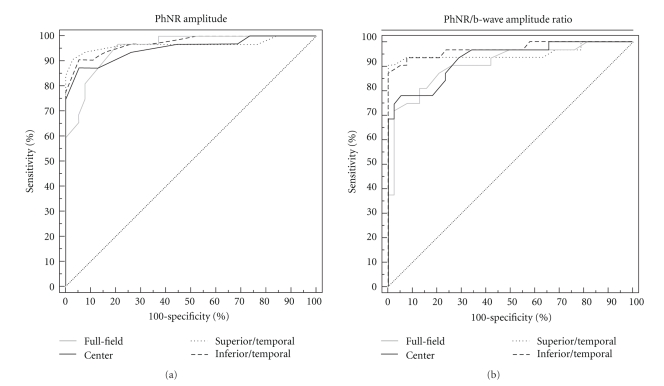
Receiver operating characteristic (ROC) curves for the PhNR amplitude (a) and PhNR/b-wave amplitude ratio (b) of the full-field and focal electroretinograms. Patients with *advanced* glaucoma (*n* = 34, mean deviation <−12 dB). PhNR: photopic negative response.

The cut-off values were varied by 1.0 *μ*V steps for the full-field PhNR amplitude, 0.1 *μ*V for the focal PhNR amplitudes, and 0.01 for the focal PhNR/b-wave amplitude ratio for the pooled data of glaucomatous and normal eyes. The sensitivity and specificity were obtained for each cut-off value and plotted to determine the ROC curves from which the AUC was obtained (Figures 5–7, Table 1).

In early glaucoma, the focal PhNR amplitude curves were always superior to the full-field PhNR amplitude curves. As a result, the AUC of the focal PhNR amplitude of the inferior/temporal area was significantly larger than that of the full-field PhNR amplitude (Figure 5(a), *P* < .05). The AUCs of the focal PhNR/b-wave amplitude ratio obtained from all retinal areas were significantly larger than those of the full-field PhNR/b-wave amplitude ratio (Figure 5(b), Table 1, *P* = .01 for the center, *P* = .001 for the superior/temporal area, and *P* < .001 for the inferior/temporal area). 

For eyes with intermediate glaucoma, most parts of the ROC curves of the focal ERG amplitudes overlapped the curve of the PhNR amplitude of the full-field ERGs. Thus, there was no significant difference in the AUCs between the focal and full-field PhNR amplitudes (Figure 6(a)). For the PhNR/b-wave amplitude ratio, the curves of the focal PhNR/b-wave amplitude ratio were always higher than those of the full-field PhNR/b-wave amplitude ratio, resulting in significantly larger AUCs for the focal PhNR/b-wave amplitude ratio than for the full-field PhNR/b-wave amplitude ratio (Figure 6(b), *P* < .05 for the center, *P* < .01 for the inferior/temporal and superior/temporal areas).

In eyes with advanced glaucoma, the ROC curves for the PhNR amplitude and PhNR/b-wave amplitude ratio of the focal and full-field ERGs were overlapped (Figure 7). The differences in the AUCs between the full-field and focal PhNRs for both the PhNR amplitude and PhNR/b-wave amplitude ratio were not significant.

### 3.4. Sensitivity and Specificity of Full-Field and Focal ERG PhNR

The sensitivity and specificity were obtained with the optimal cut-off values for the PhNR amplitude (Table 2) and the PhNR/b-wave amplitude ratio (Table 3). Because the likelihood ratio reveals the sensitivity/false positive rate, the highest likelihood ratio indicates high sensitivity and specificity. Eyes were classified as being glaucomatous when their focal PhNR amplitudes or focal PhNR/b-wave amplitude ratio were less than the cut-off values in either retinal areas (combined criterion in Tables 2 and 3). In all patient groups with different degrees of visual field defects, no significant difference was found in the specificity between the full-field and focal PhNRs obtained from all retinal areas including the combined criteria.

In patients with mild defects of the visual field, the sensitivities of the focal PhNR amplitudes were significantly higher than those of the full-field PhNR amplitudes (*P* < .01) except for the inferior/temporal area. For the PhNR/b-wave amplitude ratio, the sensitivities of the focal ERG in both retinal areas were significantly higher than those of the full-field ERGs (*P* < .001 for the center, *P* < .00001 for the superior/temporal and inferior/temporal areas). The sensitivities of the PhNR amplitude and PhNR/b-wave amplitude ratio increased to 88.1% and 97.6%, respectively, when the combined criterion was used, and they were significantly higher than the corresponding values of the full-field PhNR (*P* < .00001).

In intermediate and advanced glaucoma, the sensitivities of the focal PhNRs were generally higher than those of the full-field PhNRs. A significant difference was found between the focal and full-field PhNRs in the PhNR/b-wave amplitude ratio obtained from the superior/temporal and inferior/temporal areas in intermediate glaucoma (*P* < .01 for the superior/temporal retinal area, *P* < .05 for the inferior/temporal area). The sensitivities of the focal PhNR obtained by the combined criteria were significantly higher than those of the full-field PhNR in intermediate glaucoma (*P* < .05 for the PhNR amplitude, *P* < .005 for the PhNR/b-wave amplitude ratio). 

In advanced glaucoma, there was no significant difference in the sensitivity between the full-field and focal PhNRs.

## 4. Discussion

We compared diagnostic abilities between the full-field and focal PhNRs in detecting glaucomatous eyes. Our results demonstrated that the AUCs and sensitivities were higher for the focal PhNR than for the full-field PhNR at the early and intermediate stages of glaucoma. This suggests that the focal PhNR is a good indicator to detect the functional loss in early and intermediate glaucoma.

### 4.1. Diagnostic Ability of Full-Field and Focal PhNRs

The AUCs of the focal PhNRs were better for identifying eyes with early and intermediate glaucoma than those of the full-field PhNRs. On the other hand, there was no significant difference in the AUCs between the focal and full-field PhNRs in advanced glaucoma. When the combined criterion for the focal PhNR was used, the sensitivity increased to 88.1% and 97.6% for the focal PhNR amplitude and PhNR/b-wave amplitude ratio, respectively, even in early glaucoma, while the sensitivities for the PhNR amplitude and amplitude ratio of the full-field ERG were 38.1% and 23.8%. These findings indicate that the focal PhNR is a better indicator than the full-field PhNR in detecting functional changes in early and intermediate glaucoma. 

We selected the optimal cut-off value with the highest likelihood ratio which maximally reduces false positive cases. This then kept the specificity high for both PhNR parameters. The disadvantage of the combined criterion is that it lowers the specificity as reported although a high sensitivity was obtained [18]. However, the specificity of the PhNR of the full-field and focal ERGs could be kept over 90% by using this method to select the optimal cut-off values. Our results indicated that, even in early glaucoma, the focal PhNR had high sensitivity and specificity attained by the combined criterion. 

We have reported that a curvilinear relationship existed between the retinal sensitivity (in decibels) measured by perimetry and the focal PhNR amplitude [18]. This indicated that 3 dB loss in the retinal sensitivity is approximately associated with a fifty percent decrease in the focal PhNR amplitude at the early stage of glaucoma. In fact, the largest loss of the PhNR amplitude was seen at the early stage of glaucoma in the focal ERGs (Figures 3 and 4). On the other hand, the full-field PhNR amplitude gradually reduced with advance of glaucoma. Taken together, these findings indicate that the focal PhNR could be a better measure to detect functional abnormalities at the early stage of glaucoma than the full-field PhNR.

### 4.2. Disadvantages of Focal PhNR

It is essential that the ocular fundus is visible to be able to record the focal PhNRs reliably because the stimulus areas stimulated must be monitored during the recordings using an infrared fundus camera. It is impossible to record the focal ERG in patients with dense opacities of the ocular media, such as cataracts and vitreous opacities. Furthermore, opacities of the ocular media can produce stray-light that makes the focal stimulus larger. Therefore, we have excluded patients with clinically significant cataracts that affected vision. On the other hand, the stray-light effect is negligible for the full-field ERGs. In cases with severe opacity of the ocular media, the full-field PhNRs would be more reliable than the focal PhNR.

Intersession variability is represented by the coefficients of variation (CV = standard deviation/mean × 100), and it was higher for the focal PhNR than for the full-field PhNR [16, 18]. In addition, variations of the PhNR amplitude among individuals were greater for the focal PhNR amplitude than for the full-field PhNR amplitude [18]. However, this disadvantage of the focal PhNR can be reduced by using the amplitude ratio of the PhNR to the b-wave amplitude [18]. Therefore, the PhNR/b-wave amplitude ratio is recommended for measuring the effectiveness of the focal ERGs.

### 4.3. Limitations of the Present Study

The recording and stimulus conditions of the focal ERG were different from those of full-field ERG, which may explain why the focal PhNR was better than the full-field PhNR in diagnosing early or intermediate glaucoma. First, we set the low cut filters at 0.5 Hz and 5 Hz for the full-field and focal ERGs, respectively. The higher cut-off frequency (5 Hz) used to record the focal PhNR was necessary to eliminate the drifts in the baseline. Thus, some of the low frequency components of the PhNR were reduced as shown in monkeys [24, 25]. 

Second, the full-field ERGs were elicited by red stimuli on a blue background (R/B) while the focal ERGs were elicited by white stimuli on a white background (W/W). The R/B stimuli have been shown to be a very good combination to elicit large and reliable PhNRs [26]. Furthermore, the results of our preliminary study demonstrated that the sensitivity and specificity to discriminate glaucoma were higher for the R/B than for the W/W stimulus conditions (Machida et al., *IOVS* 2007; 48: ARVO E-Abstract 215). Thus, the stimulus conditions used in this study are more advantageous to eliciting full-field PhNRs than focal PhNRs. 

Therefore, the differences in the recording and stimulus conditions do not seem to be able to explain the current results in which the focal PhNR was more sensitive than the full-field PhNR in diagnosing early and intermediate glaucoma.

## 5. Conclusions

The results of this study indicate that the PhNRs of the full-field and focal ERGs represent functional loss of RGCs in glaucoma at different stages of glaucoma. The focal ERG has the diagnostic ability with high sensitivity and specificity in detecting glaucomatous eyes at the early and intermediate stages, especially when the combined criterion is used. There was no difference in the diagnostic value between the full-field and focal PhNRs in advanced glaucoma. Thus, the focal PhNR can be a good functional parameter to detect early or intermediate glaucoma.

## Figures and Tables

**Table 1:** Area under the curve of the PhNR amplitude and PhNR/b-wave amplitude ratio.

	PhNR amplitude		PhNR/b-wave amplitude ratio	
	AUC	95% CI	AUC	95% CI
*Early *(*n* = 41)				

Full-field ERG	0.748	0.638–0.839	0.666	0.551–0.768
Focal ERG				
Center	0.866	0.759–0.925	0.863	0.767–0.930
Sup/temp	0.863	0.767–0.930	0.886	0.795–0.947
Inf/temp	0.874	0.780–0.938	0.924	0.841–0.971

*Intermediate *(*n* = 28)				

Full-field ERG	0.865	0.758–0.937	0.789	0.670–0.880
Focal ERG				
Center	0.906	0.808–0.964	0.938	0.849–0.982
Sup/temp	0.929	0.838–0.978	0.946	0.860–0.987
Inf/temp	0.959	0.878–0.992	0.942	0.854–0.984

*Advanced *(*n* = 34)				

Full-field ERG	0.954	0.875–0.989	0.910	0.817–0.965
Focal ERG				
Center	0.951	0.871–0.988	0.930	0.842–0.977
Sup/temp	0.968	0.895–0.995	0.953	0.874–0.989
Inf/temp	0.972	0.902–0.996	0.972	0.901–0.996

PhNR: photopic negative response; AUC: area under the curve; CI: confidence interval; sup/temp: superior/temporal; inf/temp: inferior/temporal.

**Table 2:** Sensitivity and specificity of the PhNR amplitude to discriminate glaucomatous eyes.

	Sensitivity (95%CI)	Specificity (95%CI)	Cut-off value (*μ*V)
*Early *(*n* = 41)			

Full-field ERG	38.1 (23.6–54.4)	92.3 (79.1–98.3)	22
Focal ERG			
Center	69.1 (52.9–82.4)	95.2 (83.8–99.3)	0.7
Sup/temp	63.4 (46.9–77.9)	97.6 (87.1–99.6)	0.5
Inf/temp	56.1 (46.9–77.9)	95.2 (83.8–99.3)	0.7
Combined	88.1 (74.4–96.0)	90.5 (87.7–99.6)	

*Intermediate *(*n* = 28)			

Full-field ERG	59.3 (38.8–77.6)	92.3 (79.1–98.3)	22
Focal ERG			
Center	64.3 (44.1–81.3)	95.2 (83.8–99.3)	0.7
Sup/temp	75.0 (55.1–89.3)	97.6 (87.1–99.6)	0.5
Inf/temp	67.9 (47.7–84.1)	95.2 (83.8–99.3)	0.7
Combined	92.9 (87.7–99.6)	90.5 (87.7–99.6)	

*Advanced *(*n* = 34)			

Full-field ERG	66.7 (48.2–82.0)	92.3 (79.1–98.3)	22
Focal ERG			
Center	88.2 (72.5–96.6)	95.2 (83.8–99.3)	0.7
Sup/temp	90.9 (75.6–98.0)	97.6 (87.1–99.6)	0.5
Inf/temp	90.9 (75.6–98.0)	95.2 (83.8–99.3)	0.7
Combined	97.1 (87.7–99.6)	90.5 (87.7–99.6)	

PhNR: photopic negative response; CI: confidence interval; sup/temp: superior/temporal; inf/temp: inferior/temporal.

**Table 3:** Sensitivity and specificity of the PhNR/b-wave amplitude ratio to discriminate glaucomatous eyes.

	Sensitivity (95%CI)	Specificity (95%CI)	Cut-off value
*Early *(*n* = 41)			

Full-field ERG	23.8 (12.1–39.5)	97.4 (86.5–99.6)	0.19
Focal ERG			
Center	61.9 (45.6–76.4)	97.6 (87.4–99.6)	0.22
Sup/temp	75.6 (59.7–87.6)	97.6 (87.1–99.6)	0.23
Inf/temp	73.1 (57.1–85.3)	95.2 (83.8–99.3)	0.29
Combined	97.6 (87.7–99.6)	92.9 (87.7–99.6)	

*Intermediate *(*n* = 28)			

Full-field ERG	40.7 (22.4–61.2)	97.4 (86.5–99.6)	0.20
Focal ERG			
Center	67.9 (47.7–84.1)	97.6 (87.4–99.6)	0.22
Sup/temp	85.7 (67.3–95.9)	97.6 (87.1–99.6)	0.23
Inf/temp	78.6 (59.0–91.7)	95.2 (83.8–99.3)	0.29
Combined	96.4 (87.7–99.6)	92.9 (87.7–99.6)	

*Advanced *(*n* = 34)			

Full-field ERG	69.7 (51.3–84.4)	97.4 (86.5–99.6)	0.20
Focal ERG			
Center	70.6 (52.5–84.9)	97.6 (87.4–99.6)	0.22
Sup/temp	90.9 (75.6–98.0)	95.6 (87.1–99.6)	0.23
Inf/temp	90.9 (75.6–98.0)	95.2 (83.8–99.3)	0.29
Combined	97.1 (87.7–99.6)	92.9 (87.7–99.6)	

PhNR: photopic negative response; CI: confidence interval; sup/temp: superior/temporal; inf/temp: inferior/temporal.
